# Ortner’s syndrome presenting as thoracic aortic aneurysm mimicking thoracic malignancy: a case report

**DOI:** 10.1186/s13256-015-0629-1

**Published:** 2015-06-24

**Authors:** Upul Pathirana, Saman Kularatne, Sumana Handagala, Gamini Ranasinghe, Ravinda Samarasinghe

**Affiliations:** 1 Department of Respiratory Medicine, National Hospital for Respiratory Diseases, Negombo Road, Welisara, Sri Lanka; 2 Department of Thoracic Surgery, National Hospital for Respiratory Diseases, Negombo Road, Welisara, Sri Lanka; 3 Department of Cardiothoracic Surgery, National Hospital of Sri Lanka, E W Perera Mawatha, Colombo 10, Sri Lanka; 4 Department of Radiology, General Hospital (Teaching), Peradeniya Road, Kandy, Sri Lanka

**Keywords:** Hoarseness, Ortner’s syndrome, Aortic aneurysm, Thoracic malignancy

## Abstract

**Introduction:**

Ortner’s syndrome is defined as left recurrent laryngeal nerve palsy caused by a cardiovascular pathology. Here we report the case of a 68-year-old man who presented to our hospital with hoarseness, whose initial chest imaging mimicked a thoracic neoplastic process with left pleural effusion. The final diagnosis was Ortner’s syndrome due to the silent rupture of a thoracoabdominal aortic aneurysm. Diagnostic thoracentesis, before computed tomography, in resource-poor settings, may have resulted in an adverse outcome in our case.

**Case presentation:**

A 68-year-old Sri Lankan man was referred to us by an otolaryngologist for further evaluation of a suspected thoracic malignancy. His presenting symptom was hoarseness of three months duration. He had essential hypertension for the last four years and had a history of 25 pack-years of cigarettes smoking. His chest X-ray showed a left-sided mediastinal mass with mild to moderate pleural effusion. An ultrasound appeared to show an encysted pleural fluid collection. However, we proceeded with computed tomography before diagnostic thoracentesis. The diagnosis of Ortner’s syndrome was made after the computed tomography due to the silent rupture of his thoracoabdominal aortic aneurysm.

**Conclusions:**

Hoarseness due to left recurrent laryngeal nerve palsy can be the presenting symptom of cardiovascular pathologies, Ortner’s syndrome. Silent rupture of thoracic aortic aneurysms can mimic that of thoracic malignancy, which is reported in literature. We illustrate the importance of a high degree of suspicion of cardiovascular pathology in order to avoid an adverse outcome following diagnostic thoracentesis.

## Introduction

Ortner’s syndrome, or cardiovocal syndrome, is defined as left recurrent laryngeal nerve palsy caused by a cardiovascular pathology, such as left atrial enlargement, dilatation of the left pulmonary artery, or a thoracic aortic archaneurysm (TAA) [1]. Although patients with TAA are often asymptomatic, ascending and arch aneurysms can erode into the mediastinal structures, causing hoarseness due to left recurrent laryngeal nerve compression, hemi-diaphragmatic paralysis due to phrenic nerve compression, and so forth. Although the rupture is a deadly complication of TAA, a slow leak can have a chronic presentation. Here, we report the case of aruptured TAA presenting as Ortner’s syndrome, closely mimicking thoracic malignancy, which would have been associated with adverse outcome following diagnostic thoracentesis.

## Case presentation

A 68-year-old Sri Lankan man was referred by an otolaryngologist in a general hospital to a pulmonologist at our hospital for further evaluation of suspected thoracic malignancy. He had transient episodes of syncope during the last year, although his main presenting symptom was hoarseness. He was known to have had essential hypertension during the last four years and had a history of 25 pack-years of cigarettes smoking. There was no family history of malignancy or connective tissue disorders. His general examination did not reveal any clubbing of his fingers or lymphadenopathy. There was a stony dull percussion note with absent breath sounds in the left lower zone of his chest. His blood pressure was 130/100mmHg in both arms, and his cardiovascular examination was otherwise unremarkable.

**Fig. 1:**
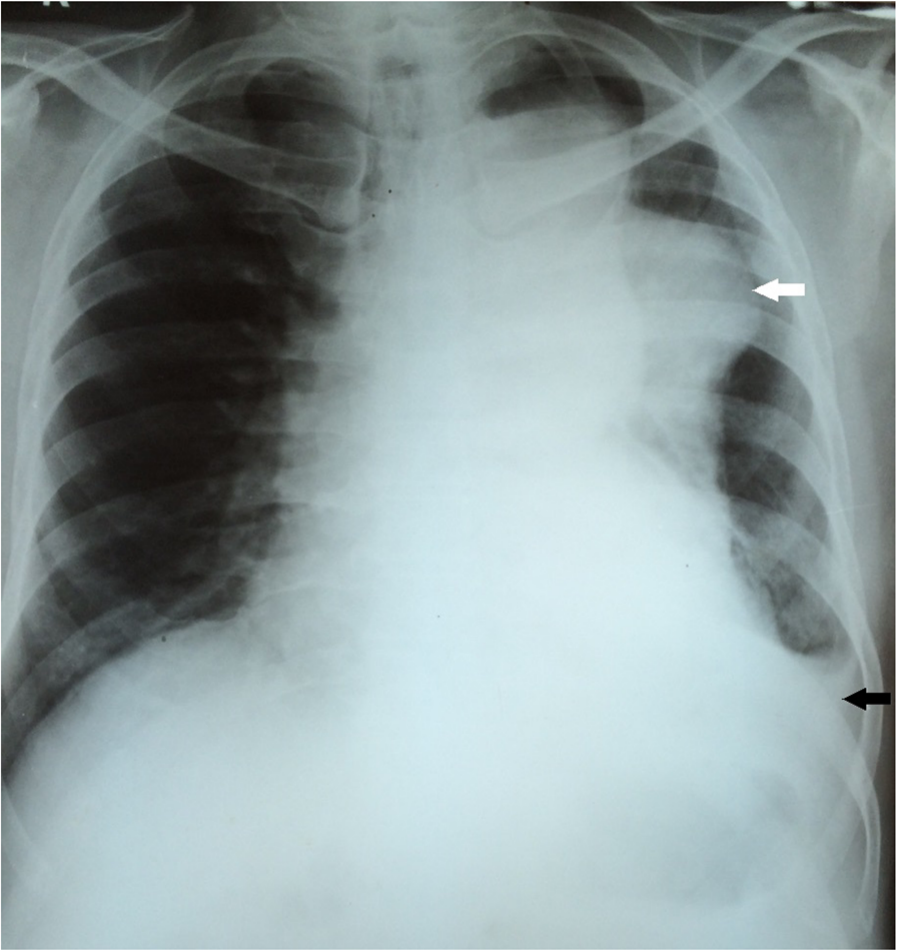
Chest X-ray showing a left side mediastinal mass. A homogeneous, soft tissue, dense, well-defined lesion in the left lung (white arrow) extending from the aortic knuckle to the left hemidiaphragm. Its medial margin merges with the mediastinum and there is a left-sided mild to moderate pleural effusion (black arrow)

An indirect laryngoscopic examination was performed by the otolaryngologist, who noted left vocal cord palsy. His chest X-ray showed a homogeneous, soft tissue, dense, well-defined lesion in his left lung, extending from the aortic knuckle to the left hemidiaphragm (Fig. 1). Its medial margin merged with the mediastinum and there was a left-sided mild to moderate pleural effusion also. An ultrasound examination of his chest revealed a cystic area in the left hemithorax with adjacent medial lung collapse. The impression was that of encysted pleural fluid collection. However, we were unable to exclude an underlying mass lesion. His transthoracic echocardiogram was normal, except for a trivial mitral regurgitation. His blood test results, including complete blood count, inflammatory markers, renal functions, liver functions, and lipid profile, were all normal or within normal limits.

**Fig. 2:**
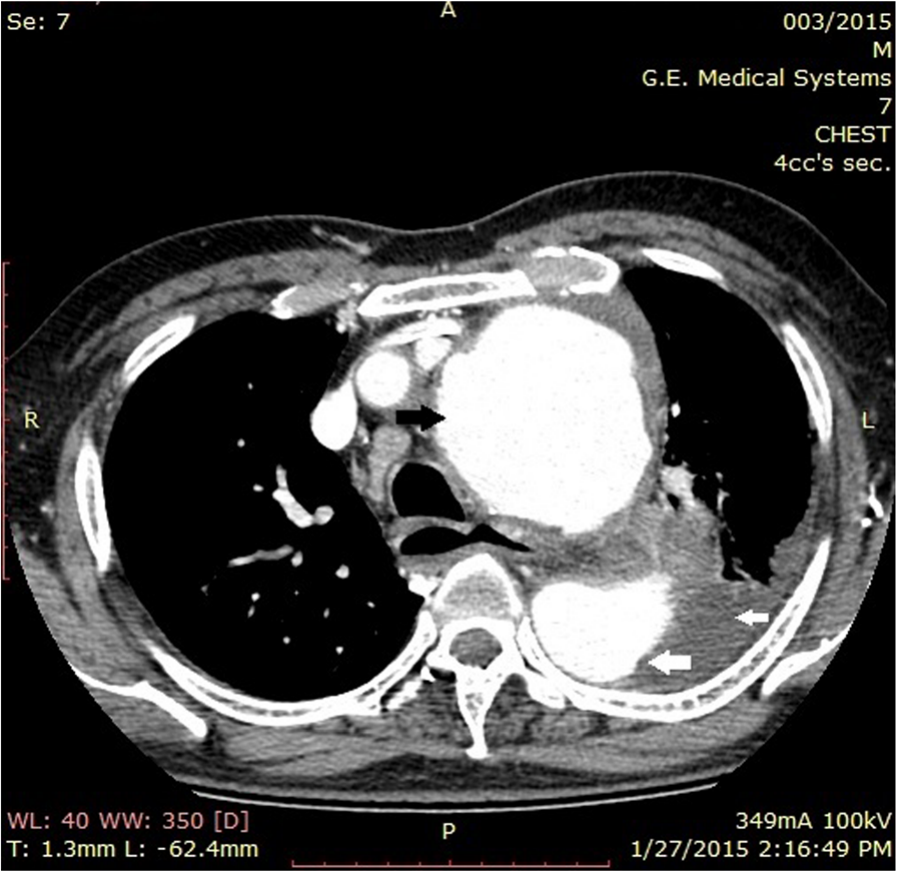
Computed tomography aortography showing ascending (black arrow) and descending (large white arrow) aortic aneurysm with left-sided pleural effusion (small white arrow)

**Fig. 3:**
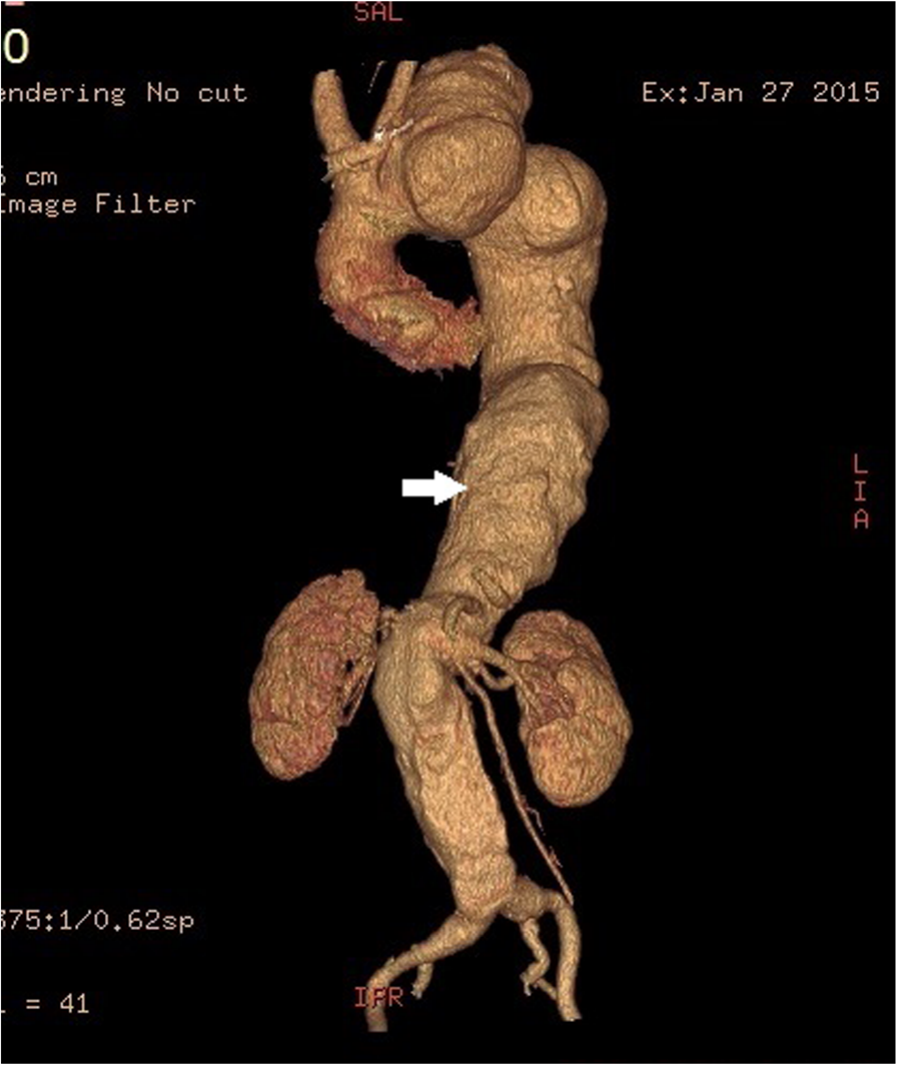
Volume-rendered image showing fusiform dilatation of the aorta (white arrow) from the aortic root to the bifurcation

His contrast-enhanced computed tomography (CT) scan with aortography showed a thoracoabdominal aortic aneurysm with peripheral thrombosis (Fig. 2). The left-sided pleural effusion was suggestive of a leaking aneurysm. A volume-rendered image of his aorta showed fusiform dilatation of the entire aorta, from aortic root to the bifurcation (Fig. 3).

The final diagnosis of Ortner’s syndrome due to the silent rupture of his thoracoabdominal aortic aneurysm was made on the basis of clinical presentation and CT aortographic appearance. The cardiothoracic surgical team attended to him and decided to manage medically without surgical intervention. Nifedipine SR 20mg was replaced by metoprolol MR 100mg daily and atorvastatin 10mg daily was added to the medical management. He is now under the care of a cardiologist for the optimum control of his atherosclerotic risk factors, and his symptoms had not progressed on his follow-up visits up to now.

## Discussion

Aortic aneurysm is defined as a permanent localized dilation of the aorta, having at least a 50 % increase in diameter compared with the expected normal diameter [2]. Abdominal aortic aneurysms are more common than aneurysms of the thoracic aorta. However, our patient had a thoracoabdominal aortic aneurysm. Although, TAAs are often asymptomatic, they are more symptomatic than abdominal aortic aneurysms. The presenting symptoms depend upon the location of aneurysm; they can compress or erode into the thoracic structures, such as neural, bony, or other soft tissues [3]. The presentation of hoarseness in our patient was due to the compression of his left recurrent laryngeal nerve, which gave the diagnosis of Ortner’s syndrome.

Thoracic malignancies, mainly bronchogenic cancers, present with hoarseness in higher frequencies (32 % of vocal cord palsy) than TAA [4]. The chest X-ray appearance can be similar in both malignancies and aneurysms, as it was in our patient. Pleural effusions in malignancies can be malignant or para-malignant, and it is frequent in respiratory wards. Though the ruptured TAA presents as a catastrophic event, slowly leaking aneurysms can have a sub-acute or chronic presentation, with pleural effusions mimicking malignancy [4].

An ultrasound scan of our patient’s chest showed what appeared to be encysted pleural fluid collection. Diagnostic thoracentesis with pleural fluid analysis gives the most accurate diagnosis in the majority of cases. As our main differential diagnoses of this patient were thoracic malignancy and silent rupture of a TAA, we preceded with a contrast-enhanced CT chest scan before diagnostic thoracentesis or bronchoscopy. His CT chest scan with aortography confirmed the diagnosis of leaking aneurysm of the thoracoabdominal aorta. A thoracentesis in this instance would have caused an adverse or fatal outcome.

Cystic medial degeneration that weakens the aortic wall results in TAA. It is normal with aging, but can be aggravated by hypertension. The risk factors that are involved in the aneurysm formation are same as those for atherosclerosis: hypertension, hyperlipidemia, and smoking [5]. Connective disorders, such as Marfan syndrome and Ehlers-Danlos syndrome, should be considered in young patients. Large artery complications like aortic aneurysm and aortic dissection are a particular problem in giant cell arteritis (GCA) [6]. The absence of features of connective tissue disorders or GCA, in combination with normal inflammatory markers, is suggestive of atherosclerosis, as was the etiology in our patient who had hypertension and smoking as main atherosclerotic risk factors.

The treatment options include surgical and medical therapy. Our patient’s advanced age, symptomatic aneurysm, and Crawford type II aneurysm increase the morbidity and mortality risk following open TAA repair [7]. As such, the cardiothoracic team decided to manage his disease conservatively, with optimum risk factor control. He was also motivated for smoking cessation. As beta blockers are shown to reduce the risk of abdominal aortic aneurysm expansion [8] and rupture, nifedipine SR 20mg twice daily was replaced with metoprolol MR 100mg daily for the control of his hypertension. As statins have been shown to be protective in cases of TAA [9], atorvastatin 10mg daily was added despite his normal lipid levels. His care was taken over by the cardiologist for follow-up.

## Conclusions

The silent rupture of a TAA can mimic that of thoracic malignancy. A CT scan is not available prior to a diagnostic thoracentesis in resource-poor settings though it is not an absolute prerequisite for thoracentesis. We illustrate the importance of the careful interpretation of chest X-rays and the clinical picture to avoid adverse outcome from diagnostic thoracentesis or intercostal tube insertion, in cases of cardiovascular pathology causing massive effusion.

## Consent

Written informed consent was obtained from the patient for publication of this case report and any accompanying images. A copy of the written consent is available for review by the Editor-in-Chief of this journal.

## Abbreviations

CT: Computed tomography
GCA: Giant cell arteritis
TAA: Thoracic aortic aneurysm
